# Occurrence, Distribution and Toxins of Benthic Cyanobacteria in German Lakes

**DOI:** 10.3390/toxics11080643

**Published:** 2023-07-25

**Authors:** Franziska Bauer, Immanuel Wolfschlaeger, Juergen Geist, Jutta Fastner, Carina Wiena Schmalz, Uta Raeder

**Affiliations:** 1 Aquatic Systems Biology Unit, Limnological Research Station Iffeldorf, Technical University of Munich, Hofmark 1–3, 82393 Iffeldorf, Germanyuta.raeder@tum.de (U.R.); 2 German Environment Agency, Schichauweg 58, 12307 Berlin, Germany

**Keywords:** cyanobacteria, cyanotoxins, benthic, planktic, lakes, anatoxin, microcystin, LC-MS/MS, ELISA, MiSeq, public health

## Abstract

Cyanobacteria are favored by climate change and global warming; however, to date, most research and monitoring programs have focused on planktic cyanobacteria. Benthic cyanobacteria blooms also increase and pose a risk to animal and human health; however, there is limited knowledge of their occurrence, distribution and the toxins involved, especially in relation to their planktic conspecifics. Therefore, we analyzed the benthic and planktic life forms of cyanobacterial communities in 34 lakes in Germany, including a monitoring of cyanotoxins. Community analyses were based on microscopic examination and Illumina sequencing of the 16S rRNA gene. The analyses of cyanotoxins were carried out using LC-MS/MS and ELISA. Observed benthic mats containing cyanobacteria consisted mainly of *Nostocales* and *Oscillatoriales*, being present in 35% of the lakes. Anatoxin was the most abundant cyanotoxin in the benthic samples, reaching maximum concentrations of 45,000 µg/L, whereas microcystin was the predominate cyanotoxin in the open-water samples, reaching concentrations of up to 18,000 µg/L. Based on the results, specific lakes at risk of toxic cyanobacteria could be identified. Our findings suggest that monitoring of benthic cyanobacteria and their toxins should receive greater attention, ideally complementing existing open-water sampling programs with little additional effort.

## 1. Introduction

Cyanobacteria can be found worldwide. In surface waters, they either occur in the pelagial, i.e., in the open water and belong to the phytoplankton, or they are part of the periphyton and grow as benthic cyanobacteria on the bottom of water bodies where they colonize various substrates such as different types of sediment or macrophytes [1].

All cyanobacteria perform photosynthesis and contribute substantially to the global oxygen production and carbon fixation [2]. Under eutrophic conditions, i.e., nutrient enrichment, certain species can proliferate excessively and form so-called water blooms. Especially among the bloom-forming species, there are strains that produce toxins that can pose a threat to humans and the aquatic environment [3].

In recent years, cyanobacterial blooms have become more frequent in Europe [4]. While the primary cause of mass development of planktic cyanobacteria is eutrophication, climate change may further increase the abundance of cyanobacteria [5,6,7,8]. The two major reasons for this are rising water temperatures and the changing stratification of lakes [5,6,9,10,11]. In addition to planktic cyanobacteria, the occurrence of benthic cyanobacteria has been increasingly reported in recent years [12,13]. Some representatives of the benthic cyanobacteria can produce the highly neurotoxic anatoxins that have led to poisoning in animals [14,15,16]. Benthic cyanobacteria are also increasingly detected in Germany, often as a result of the search for the cause of dog intoxications [17,18,19]. To date, however, very little is known about the reasons for the observed increasing abundance of benthic cyanobacteria, and there are no systematic monitoring data on their distribution and toxin production, at least not in Germany.

Planktic cyanobacteria occur in the lighted (euphotic) zone of shallow and deeper lakes. Due to reflections at the water surface and the absorption of light by various water constituents and algae, the light attenuates rapidly in deeper layers of nutrient-rich lakes, so that the deeper zone is often aphotic and therefore cannot be colonized by photosynthetically active cyanobacteria. However, there are also more nutrient-poor waters where light can penetrate to great depths. The bottom of these lakes is expected to provide the preferred habitat for benthic cyanobacteria. So far, however, only few data are available on this issue in Europe. However, for example, toxic benthic cyanobacteria have been found in nutrient-poor mountain lakes in Switzerland [20]. Other records of benthic cyanobacteria have recently been reported from the mesotrophic Lake Tegel and Reservoir Mandichosee in Germany [17,18]. The patchiness of existing data on the distribution of benthic cyanobacteria and the lack of knowledge concerning their toxin production make it difficult to assess their potential threats to humans and aquatic biota.

The objective of this study was to investigate different lake types in Bavaria (Germany) with respect to the presence of benthic and planktic cyanobacteria. Of particular interest were (i) the occurrence of benthic cyanobacteria and their geographical distribution, and (ii) the identification and quantification of the cyanotoxins produced by benthic cyanobacteria. A total of 34 water bodies were examined for the occurrence of benthic, as well as planktic cyanobacteria in 2021 and 2022.

In addition to recording the cyanobacterial community compositions by microscopical analyses and Illumina sequencing of 16S rRNA genes, cyanotoxins were determined. The emphasis was on the most common cyanotoxins anatoxins (ATXs), cylindrospermopsins (CYNs), microcystins (MCs) and saxitoxin (STX). Analyses of cyanotoxins were carried out using LC-MS/MS (ATXs, CYNs, MCs) and ELISA (STX).

We hypothesized (i) that benthic cyanobacteria occur predominantly in nutrient-poor waters and (ii) that benthic cyanobacteria and their toxins differ from planktic cyanobacterial communities with respect to the composition of genera and the cyanotoxins produced.

## 2. Materials and Methods

### 2.1. Study Site

**Figure 1:**
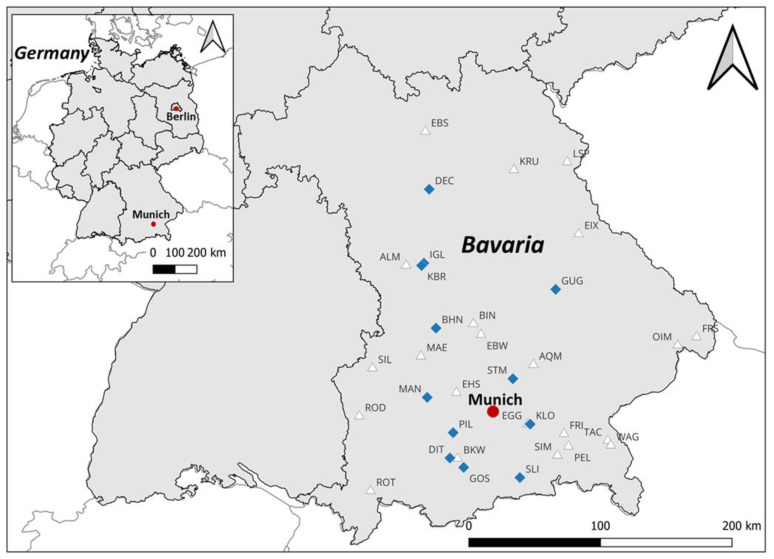
Map of all 34 sampled water bodies in Bavaria (Germany). Blue rhomb: water bodies with findings of benthic cyanobacteria. White triangle: water bodies without benthic cyanobacteria findings. Planktic cyanobacteria were found in all studied lakes. A list of lake names and their abbreviations can be found in the Supplementary Materials (Table S1). Built with QGIS (http://www.qgis.org (accessed on 5 January 2023)), data/maps copyright: Geofabrik GmbH and OpenStreetMap Contributors (https://download.geofabrik.de/europe/germany/bayern.html (accessed on 5 January 2023)).

The study comprised 34 different water bodies in Bavaria (Germany). They were selected based on consultation with all 17 Bavarian water management offices and the Bavarian Health and Food Safety Authority (LGL). The participating agencies provided information on recent cyanobacterial occurrences and indicated lakes in their area of responsibility that might be of interest for the study. However, these background data were limited to the observed occurrence of planktic cyanobacteria. Only in one of the lakes (Reservoir Mandichosee, MAN) has the occurrence of benthic cyanobacteria already been documented [18,19]. The map (Figure 1) shows all 34 water bodies which were sampled during the study. A list of lake names and their abbreviations can be found in the Supplementary Materials (Table S1).

### 2.2. Sampling Strategy

Both the planktic cyanobacteria of the open water and the benthic cyanobacteria were assessed. Open-water samples were taken in every lake, and benthic samples were taken when macroscopically visible benthic biofilm growth was present in the littoral area. Particular attention was paid to growth on different substrates, algal mats floating at the water surface, and aquatic plants washed up. Sampling was performed from the shore. Samples from the open water with a volume of 1 L were taken from 0.2 m water depth using a sampling container attached to a stick and stored in sterile water bottles. Benthic samples were collected by hand using gloves. In many cases the cells were collected with their sediment and the samples were further processed in the laboratory using forceps. The benthic samples were transported to the laboratory in sterile 50 mL falcons or sterile Tupperware jars with lids. In the search for benthic cyanobacteria, the area accessible from the shore of each studied lake was searched. A total of 130 samples were taken, 38 benthic and 92 open-water samples.

The sampling period extended from June 2021 to March 2022. Water samples were collected for microscopic and molecular genetic analyses, for toxin analysis as well as for hydrochemical measurements. When collecting benthic material, the focus was on including as much of the putative cyanobacterial mats in as little lake water as possible. All samples were cooled during transport and directly processed or frozen at −20 °C upon arrival at the laboratory.

### 2.3. Microscopy

After sampling, all unpreserved samples were investigated microscopically (Leica DMRBE, Leica, Wetzlar, Germany). The microscope is equipped with a photographic device (Zelos 285 GV, Kappa, Norderstedt, Germany) and allowed photographic documentation.

Cells in the open-water samples were concentrated by filtration for microscopic analysis, while cells in the benthic samples were collected directly using a sterile inoculation loop. Microscopic observation provided preliminary information on the phytoplankton composition and cyanobacterial communities of the samples.

### 2.4. DNA Extraction and Illumina MiSeq Sequencing

For DNA extraction, the cells were concentrated by filtering open-water samples through 0.2 µm cellulose nitrate filters (Sartorius, Goettingen, Germany). The filtrate was discarded, and the filters were frozen at −20 °C until further processing. Cells from the benthic samples were transferred to Eppendorf tubes using a sterile inoculation loop and also frozen at −20 °C until further use. Subsequently, DNA extraction was performed using the phenol-chloroform-based method described by Zwirglmaier et al. [21]. The DNA extraction of cells from benthic and open-water samples followed the same protocol.

Illumina MiSeq sequencing was used to analyze the cyanobacterial communities of benthic and open-water samples. Sequencing was performed by LGC Genomics, Berlin (Germany). The samples were sequenced bidirectionally.

The following primer pair was used for the initial PCR: S-D-Bact-0341-b-S-17 (5′ *TCGTCGGCAGCGTCAGATGTGTATAAGAGACAG*CCTACGGGNGGCWGCAG 3′) and S-D-Bact-0785-a-A-21 (5′ *GTCTCGTGGGCTCGGAGATGTGTATAAGAGACAG*GACTACHVGGGTATCTAATCC 3′) [22]. Illumina adaptors are shown in italics. These primer pairs cover the variable regions V3–V4 of the 16S rRNA gene. Quality control and raw data analysis were also performed by LGC Genomics. Classification was carried out based on the SILVA taxonomy [23]. If the classification was not clear, a subsequent analysis was carried out using the NCBI nucleotide blast [24].

#### Enzyme-Linked Immunosorbent Assay

ELISA test kits from Abraxis (Sension, Augsburg, Germany) were used for the quantification of the cyanotoxin STX. The procedure was performed according to the manufacturer’s instructions.

### 2.5. Analytical Detection Method (Liquid Chromatography Coupled with Mass Spectrometry, LC-MS/MS)

ATXs, CYNs and MCs were analyzed by LC-MS/MS consisting of an Agilent 2900 Series HPLC (Agilent Technologies, Waldbronn, Germany) coupled to a 5500 QTrap mass spectrometer (AB Sciex, Framingham, MA, USA) with a Turbo-Ion Spray Interface.

For the determination of ATXs and CYNs, 10 µL of the extract was separated on an Atlantis C18 column (2.1 mm, 150 mm, Waters, Eschborn, Germany) at 30 °C, a flow rate of 0.25 mL/min using a linear gradient of 0.1% formic acid (A) and 0.1% formic acid in methanol (B) [17]. Identification and quantification of ATXs (anatoxin-a, ATX; dihydroanatoxin-a, Dh-ATX; homoanatoxin-a, HATX) and CYNs (cylindrospermopsin, CYN; deoxy-cylindrospermopsin, dCYN) were performed in the “multiple reaction monitoring” (MRM) mode using the characteristic transitions as described in [17]. Standards for calibration were from Novakits (Nantes, France). Limits of detection for the anatoxin and cylindrospermopsin variants were 0.03 µg/L, and limits of quantitation were 0.1 µg/L.

MCs were separated using a Purospher STAR RP-18 column (30 mm × 4 mm, 3 mm particle size, Merck, Germany) with endcapping at 30 °C [25]. The mobile phase consisted of 0.5% formic acid (A) and acetonitrile containing 0.5% formic acid (B) with a flow rate of 0.5 mL/min with a linear gradient. The injection volume was 10 µg/L. MCs ([Asp3]-MC-RR, MC-RR, MC-YR, [Asp3]-MC-LR, MC-LR, MC-LW, MC-LF, MC-LA) were identified and quantified in MRM mode using characteristic transitions as described in Krienitz et al. [25]. Standards for calibration were from Novakits (Nantes, France). Limits of detection for each microcystin variant ranged from 0.06–0.4 µg/L, and limits of quantitation ranged from 0.2–1.2 µg/L.

### 2.6. Hydrochemical Analyses

Total phosphorus (TP) concentration was measured using the unfiltered water samples. Values of TP were determined following established standard methods by the German Chemists’ Association [26].

## 3. Results

### 3.1. Occurrence and Distribution of Benthic Cyanobacteria in the Study Area

**Figure 2:**
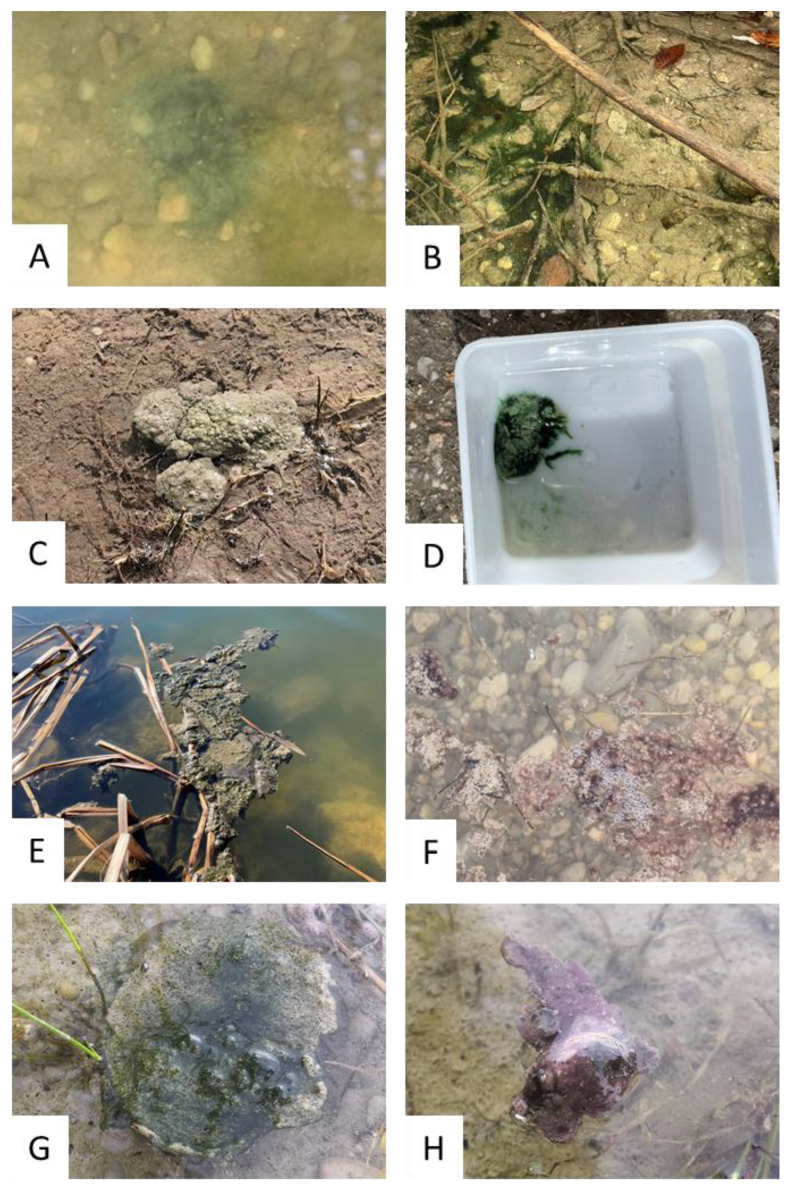
Macroscopic appearance of different benthic biofilms sampled during the study. (**A**) GUG (13 July 2021, dominated by *Neolyngbya*), (**B**) GOS (3 November 2021, dominated by *Oscillatoria*), (**C**) BHN (14 September 2021, dominated by *Oscillatoria*), (**D**) DIT (21 June 2021, dominated by *Oscillatoria*), (**E**) DEC (22 March 2022, unidentified cyanobacteria), (**F**) MAN (27 July 2021, dominated by *Tychonema*), (**G**) STM (25 August 2021, unidentified cyanobacteria), (**H**) STM (25 August 2021, dominated by *Kamptonema*). Identification of genera based on microscopy and sequencing results. A list of lake names and their abbreviations can be found in the Supplementary Materials (Table S1).

Each sampling site was first analyzed for the presence of benthic cyanobacteria based on macroscopic examination. Macroscopic appearances of benthic mats showed a great variability with respect to color and texture (Figure 2). Benthic cyanobacteria occurred mainly growing on sediment (Figure 2A–D) or as floating aggregations (Figure 2E–H). The coloration of the mats was mainly greenish or reddish-purple.

**Figure 3:**
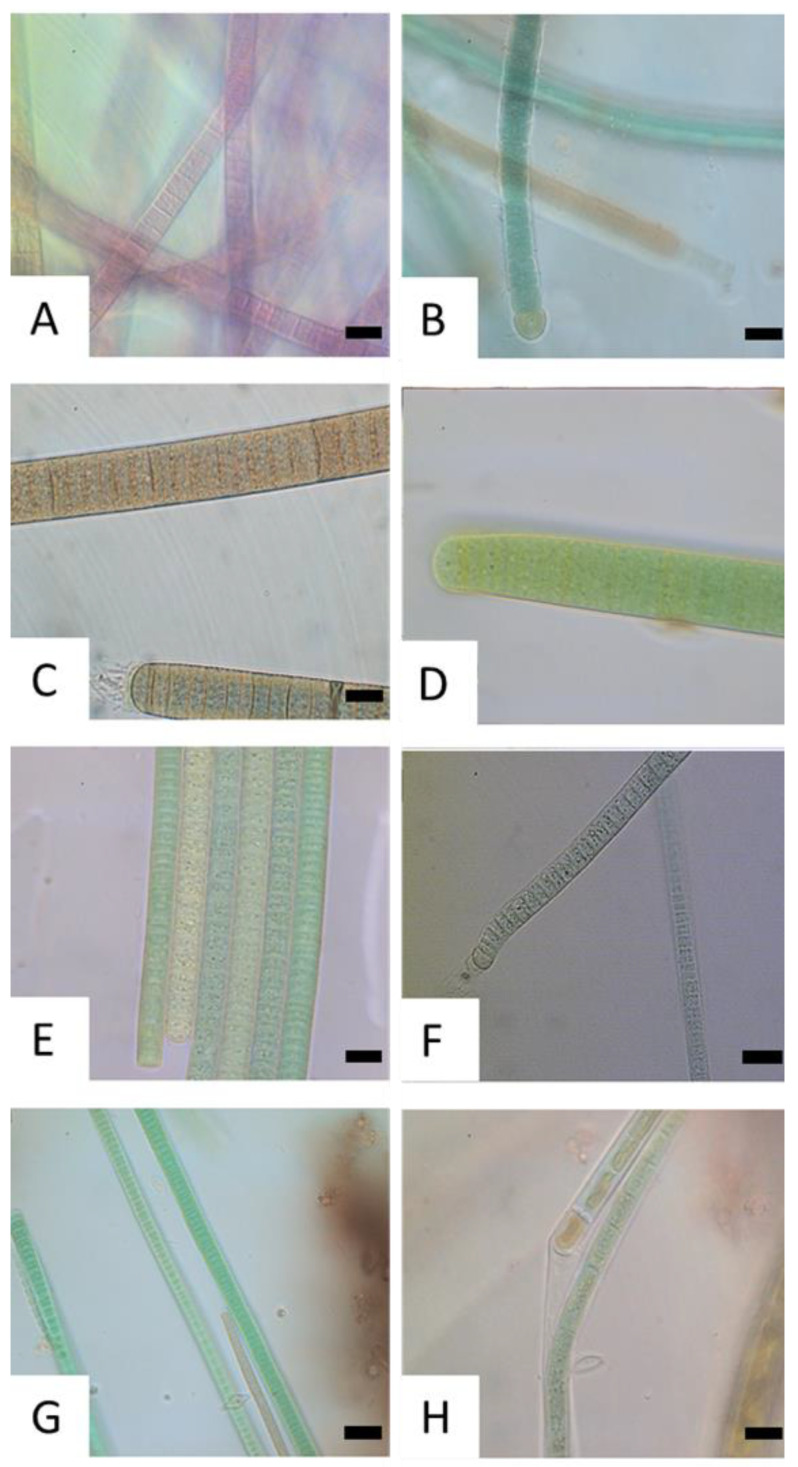
Microscopic image of different sampled benthic biofilms. Cyanobacteria of the orders Oscillatoriales and Nostocales dominate. 1000× magnification, bar = 10 µm. (**A**) MAN (27 July 2021, *Tychonema*), (**B**) KLO (29 September 2021, *Calothrix*), (**C**) KBR (23 February 2023, *Phormidium*), (**D**) STM (25 August 2021, *Oscillatoria*), (**E**) DIT (6 September 2021, Oscillatoriales), (**F**) DIT (21 June 2021, *Microcoleus*), **G**) GOS (3 November 2021, green filaments: *Oscillatoria,* red filaments: *Tychonema*), (**H**) DEC (21 September 2021, *Oscillatoria* (below), green algae (above)). Identification based on morphological characteristics. A list of lake names and their abbreviations can be found in the Supplementary Materials (Table S1).

After the sampling of visible mats, microscopic analyses were performed to confirm the presence of cyanobacteria and to exclude samples containing only algae (Figure 3). In most of the benthic mats, cyanobacteria could be detected. These were all filamentous and cyanobacteria of the orders Nostocales and Oscillatoriales dominated (Figure 3A–G). Only in very few cases, the sample consisted exclusively of green algae, which were not further analyzed and documented (Figure 3H).

Benthic cyanobacteria were detected in 12 of the 34 sampled waters, corresponding to 35% of the lakes. Planktic cyanobacteria were present in all lakes studied. The map (Figure 1) shows all sampled water bodies in Bavaria (Germany) and indicates in which lakes benthic cyanobacteria were found during the study period.

Most lakes (58%) in which benthic cyanobacteria occurred were oligotrophic, 25% of the lakes with positive records of benthic cyanobacteria were classified as oligo-mesotrophic and 16% as eutrophic.

**Figure 4:**
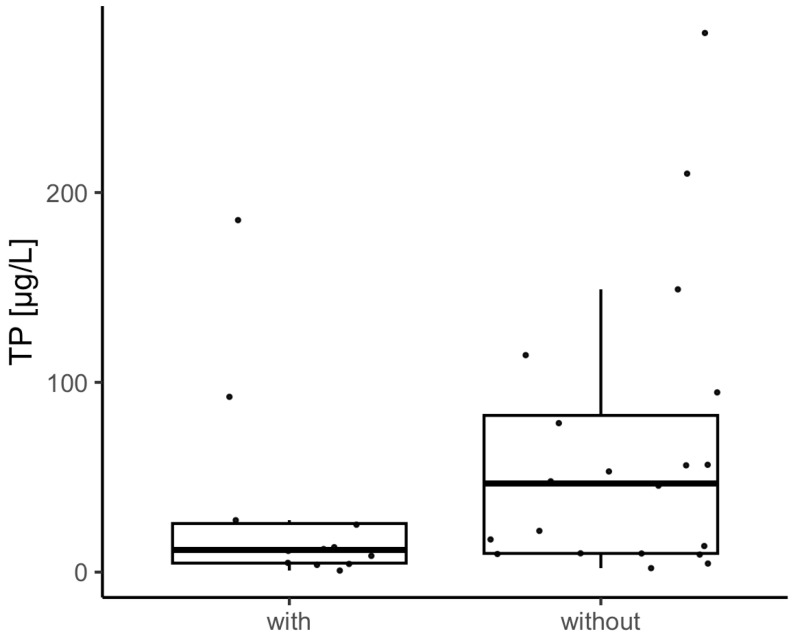
Lakes with and without benthic cyanobacteria findings and the lakes’ total phosphorus content (TP). Dots represent individual lakes.

Figure 4 illustrates that benthic cyanobacteria were predominantly found in waters with a lower TP (median 12 µg/L) content, whereas the nutrient content in lakes without benthic findings was higher (median 47 µg/L).

### 3.2. Community Composition

Samples where benthic cyanobacteria could be confirmed by microscopy were subsequently sequenced. Altogether, 37 taxa were detected in benthic samples (Table 1), of which 34 could be classified to the genus level.

In five cases, taxa could only be classified to the order level (*Caenarcaniphilales*) or to the family level (*Leptolyngbyaceae*, *Microcystaceae*, *Phormidiaceae* and *Pseudanabaenaceae*). All taxa that accounted for less than 2% of the sequences were grouped under “Other”. The most abundant genus was *Cyanobium*, which was present in 92% of benthic samples. Other common taxa which were present in at least 25% of the water bodies were the following: *Leptolyngbyaceae* (in 50% of the studied lakes), *Pseudanabaena* and *Tychonema* (in 33% of the studied lakes each), *Calothrix*, *Kamptonema*, *Leptolyngbya*, *Nodosilinea*, *Oscillatoria* and *Planktothrix* (in 25% of the studied lakes each). The total number of benthic genera in each lake ranged between three (STM, IGL) and eleven (KBR). Dominating benthic taxa in the different lakes were *Cyanobium* (DEC, KBR), *Cyanomargarita* (SLI), *Kamptonema* (STM), *Leptolyngbya* (KLO), not further classified *Leptolyngbyaceae* (IGL), *Neolyngbya* (GUG), *Oscillatoria* (DIT, GOS), *Pleurocapsa* (PIL), and *Tychonema* (MAN). In one of the samples, the sequences grouped under “Other” account for the dominant proportion (BHN). Altogether, 23 of the 37 benthic taxa were exclusively detected in benthic samples (Table 1 and Table S2).

A total of 14 genera were detected in the open-water samples (Table S2). Classification down to genus level was possible in all cases. The most common genera in the open-water samples included *Cyanobium* (in 100% of the studied lakes), *Aphanizomenon* and *Microcystis* (in 67% of the studied lakes each). The total number of planktic genera in each lake ranged between three (GUG, IGL, KLO) and eight (MAN). Dominating genera in the open-water samples were *Aphanizomenon* (DIT, KLO), *Cyanobium* (PIL, SLI, GUG, IGL), *Planktothrix* (DEC) and *Tychonema* (MAN). In contrast, 4 of the 14 planktic taxa were only detected in the open-water samples: *Aliterella*, *Cuspidothrix*, *Limnoraphis* and *Woronichinia*. In all but one of the studied lakes, the dominant genera differed in the benthic and open-water samples. Only in MAN, *Tychonema* dominated in both sample types.

### 3.3. Cyanotoxins

Both total cyanotoxins and dissolved cyanotoxins were determined for each sample. With few exceptions, the dissolved toxin values determined in the filtrate of each sample were below the detection limit. Dissolved toxins are often difficult to detect, as they are immediately diluted by the large water volume of the lake, especially at low concentrations, and are subsequently no longer detectable. Therefore, in the following, only the total toxin values are given. A table showing all the results of the toxin measurements is provided in the Supplementary Materials (Table S3).

**Figure 5:**
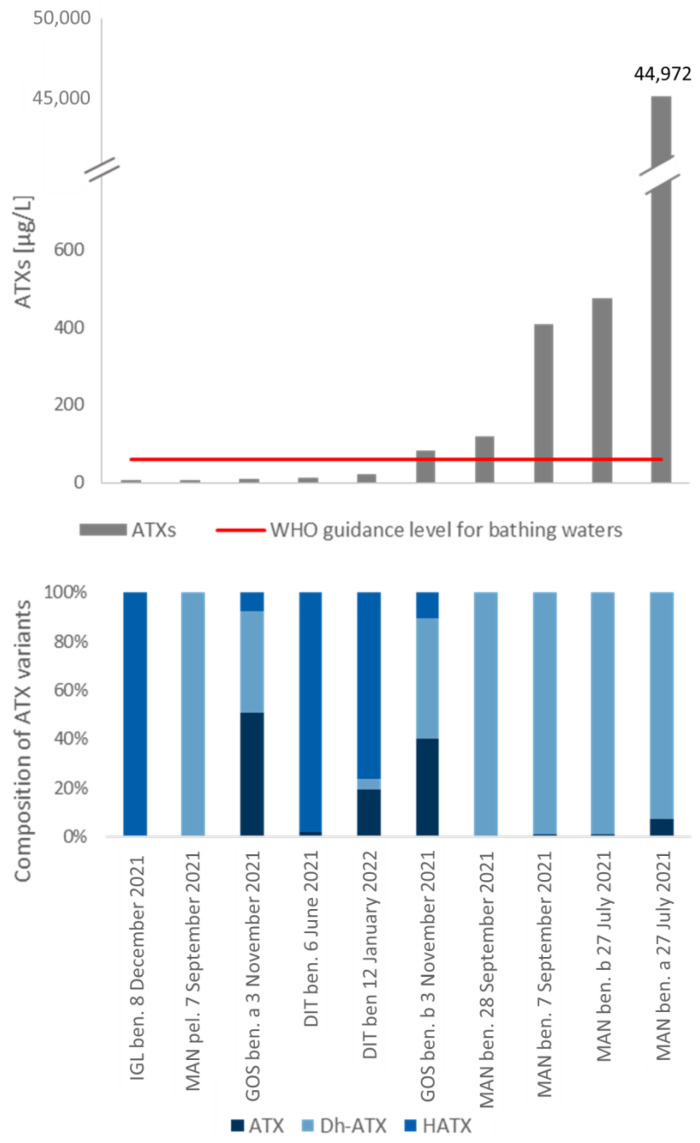
Results of anatoxin measurements based on the LC-MS/MS method. Total toxin values are shown. Above: sum of anatoxin variants (ATXs) in the samples with the ten highest ATX values measured during the study. The WHO guidance level for bathing waters for anatoxin is indicated as a red line (60 µg/L). Below: detailed composition of anatoxin variants of the samples above. ATX: anatoxin-a, Dh-ATX: dihydroanatoxin-a, HATX: homoanatoxin-a. Ben.: benthic, pel.: planktic. A list of lake names and their abbreviations can be found in the Supplementary Materials (Table S1).

The samples with the ten highest values of ATXs are shown in Figure 5. The highest ATXs value of 44,972 µg/L has been detected in one of the benthic samples of MAN on 27 July 2021. ATXs here were composed of 99.8% (44,900 µg/L) Dh-ATX and 0.2% (72 µg/L) ATX. HATX was not detected in the samples of MAN. Overall, the four samples richest in ATXs were from this lake (Figure 5). Only one of the ten samples with the highest ATXs values in the study was planktic. The measured ATXs in this sample was 4 µg/L and is therefore far below the guideline value of 60 µg/L recommended by the WHO. Comparatively high ATXs values were also determined in benthic samples from DIT and IGL. ATXs could also be detected in an open-water sample from MAN from 9 July 2021.

**Figure 6:**
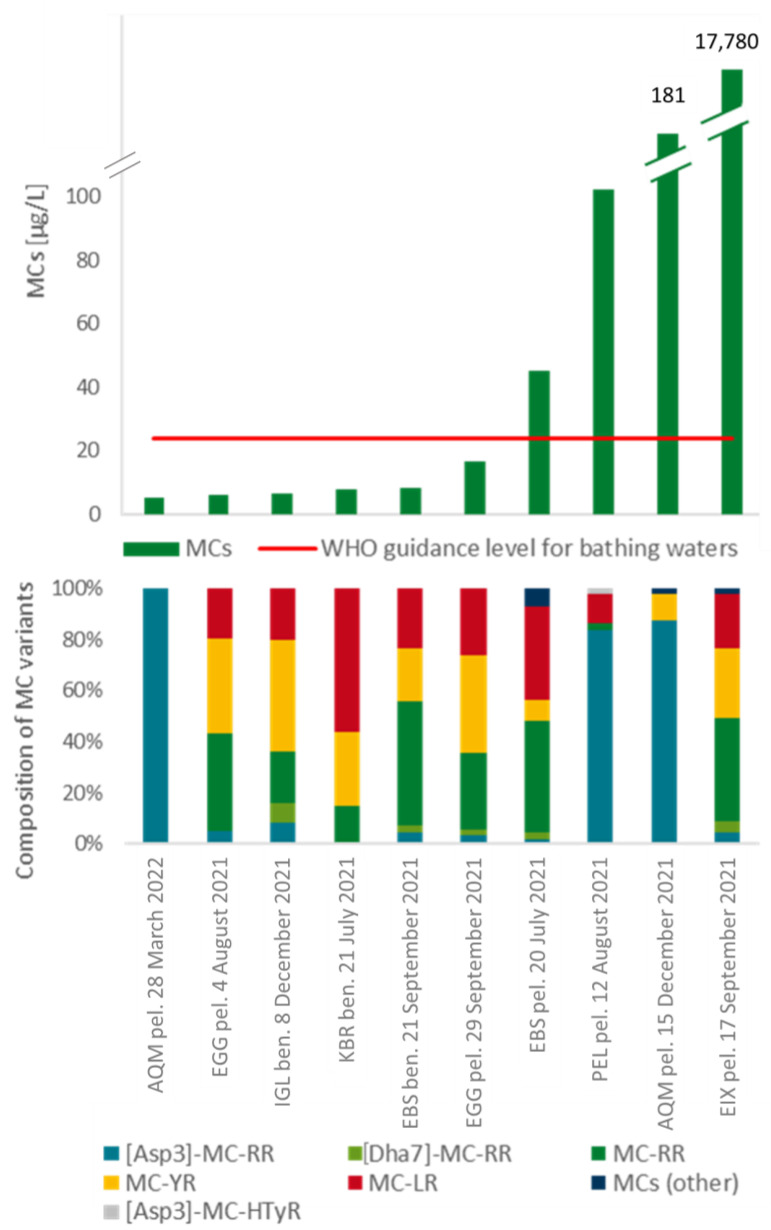
Results of microcystin measurements based on the LC-MS/MS method. Total toxin values are shown. Above: sum of microcystin variants (MCs) in the samples with the ten highest MCs values measured during the study. The WHO guidance level for bathing waters for microcystin is indicated as a red line (24 µg/L). Below: detailed composition of microcystin variants of the samples above. MC: microcystin. Ben.: benthic, pel.: planktic. A list of lake names and their abbreviations can be found in the Supplementary Materials (Table S1).

The samples with the ten highest values of MCs are shown in Figure 6. The maximum MCs value of 17,780 µg/L was measured in a sample from EIX taken on 17 September 2021. A *Microcystis* surface bloom was detected at the sampling date. The value exceeds the WHO guideline value for bathing waters (24 µg/L) more than 700-fold. The MCs components were mainly composed of MC-RR, MC-YR, and MC-LR. A very high MCs value was also measured in AQM in an open-water sample from 15 December 2021. The MCs value in this case was 182 µg/L, dominated by [Asp3]-MC-RR. Here, the actual MC producer was very likely the genus *Planktothrix rubescens*, which established a surface bloom at that time in this lake.

The WHO recommended guideline maximum value for microcystin in bathing waters of 24 µg/L was exceeded in four lakes and samples (EIX, AQM, PEL, EBS). All four samples were from the open water and in none of the corresponding lakes benthic cyanobacteria could be detected.

There are two sample types which differ in the composition of MCs. In samples from the AQM and PEL, the MCs mainly consisted of [Aps3]-MC-RR, in all other samples the MCs were mainly composed of MC-RR, MC-YR and MC-LR.

The analyses of CYNs were performed by LC-MS/MS. All results were negative. STX was analyzed by ELISA. Almost all samples were without findings, or the toxin concentrations were only very slightly above the detection limit of 0.02 µg/L. The highest STX concentration of 0.59 µg/L was measured in a benthic sample from DEC (21 September 2021), and the highest open water STX concentration of 0.35 µg/L was also found in DEC (21 September 2021). We also examined the possibility of using an Anatoxin-a ELISA as an alternative to LC MS/MS toxin analysis. The common anatoxin variant Dh-ATX was not detectable with this method and the ELISA was therefore considered unsuitable for reliable environmental analysis of anatoxins.

## 4. Discussion

The findings of this study show that benthic cyanobacteria are widespread in Bavarian lakes, and that they can produce toxins of concern—both aspects are currently insufficiently considered in existing monitoring programs which have a focus on planktic cyanobacteria. The hypothesis that benthic cyanobacteria occur predominantly in nutrient-poor waters was confirmed, stressing that problematic cyanobacterial occurrences and blooms are not restricted to water bodies with known eutrophication problems. The observed differences in the community compositions and toxins of benthic versus planktic cyanobacteria identified in our study suggest that a comprehensive monitoring approach should consider sampling of both forms in the future.

A possible explanation for the finding of benthic cyanobacteria predominantly in nutrient-poor waters is the fact that low nutrient concentrations in the water bodies generally limit the development of phyto- and zooplanktic organisms in the pelagial. As a result, a constant high amount of photosynthetic active radiation reaches the bottom of such water bodies. This provides improved growth conditions for benthic cyanobacteria [12,27]. The different substrates colonized and the spatial proximity to sediments and pore water likely influence benthic cyanobacterial growth to a larger degree than planktic representatives [28,29]. It is possible that some representatives of benthic cyanobacteria may be less dependent of the nutrient content of the water body, since they might more easily take up nutrients from the sediment they colonize, e.g., from upwelling pore water or P-release related to Fe-reduction in anaerobic sediment boundary layers [30]. A frequent occurrence of *Cyanobium* was observed in the benthic samples. However, the benthic and planktic representatives of *Cyanobium* have been assigned to the same OTUs (operational taxonomic units) based on sequencing data. It is therefore uncertain whether this is truly a benthic form or whether the detection was due to a mingling of the benthic samples with high abundant planktic cyanobacteria. In the literature, *Cyanobium* (formerly *Synechococcus*) is described as a representative of the picoplankton of marine and freshwater ecosystems and as a representative in benthic biofilms [31,32,33,34].

Based on the composition of cyanobacterial genera detected in the samples, different cyanotoxins could theoretically be produced by benthic cyanobacteria in the lakes. Several of the benthic genera such as *Geitlerinema*, *Leptolyngbya*, *Oscillatoria Planktothrix* and *Tychonema* have been shown to produce MCs [20,35,36]. A benthic MCs-producing *Planktothrix* species led to the fatal poisoning of a dog on a river in New Zealand back in 2008 [37]. The genus *Cyanomargarita* has recently been separated from the genus *Rivularia* [38], which is also a possible MCs producer [39]. *Scytonema* was also detected in the samples as a possible STX producer [40]. Moreover, the occurring genus *Dactylothamnos* is closely related to the genus *Tolypothrix*, which is probably a producer of STX [41]. As potential producers of ATX, the genera *Kamptonema*, *Microcoleus*, *Oscillatoria* and *Tychonema* could be detected [42,43,44,45], whereas *Microcoleus* and *Tychonema* are also capable of producing the variant Dh-ATX. The genus *Kamptonema* can also produce CYNs [46]. In addition, genera capable of producing yet-unidentified toxins were present in the samples. These include *Calothrix*, *Cyanothece*, *Geitlerinema* and *Leptolyngbya* [47,48,49,50,51,52]. It has not been analyzed if these genera were producing these compounds in the studied lakes. Nothing is known about the toxicity of *Neolyngbya* and *Scytolyngbya*. Geosmin is a terpene alcohol that causes the typical odor of soils and is produced by many cyanobacteria [53]. The following genera were detected as possible geosmin producers: *Kamptonema*, *Microcoleus* and *Tychonema* [54,55,56]. One cause of dog deaths associated with ATXs and *Tychonema* [17,18,19] could be the production of geosmin, which is attractive to dogs and thus leads to a fatal attraction for them [14].

In summary, based on the cyanobacteria observed in the benthic samples, it is possible that ATXs, CYNs, MCs, and STX are present in the samples. In the past, fatal poisonings of animals by ATXs and MCs produced by benthic cyanobacteria have been reported several times. In this context, the genera *Microcoleus*, *Oscillatoria*, *Planktothrix*, *Phormidium*, and *Tychonema* have been specifically cited as producers [14,17,18,37]. In our dataset, the cyanotoxins ATXs, MCs and STX were detected in different open-water samples and benthic samples. ATXs were more often dominant in the benthic samples, while MCs were mainly detected in open-water samples. STX measured by ELISA reached the maximum value in a benthic sample at a concentration of 0.6 µg/L. The STX producer could not be determined in this sample. All measured values for SXT were far below the 30 µg/L given by WHO as a guideline threshold value for bathing waters. When assessing toxicity in relation to the WHO guideline values, it must be considered that these limits for cyanotoxins are based on unintentional water intake by children during bathing (approx. 250 mL per day) and are therefore only valid for open-water samples.

Important anatoxin variants include ATX, HATX, and Dh-ATX, which were detected during the study. Previous studies suggest that Dh-ATX is about ten times less toxic than ATX [57,58]. In contrast, however, a more recent study shows that oral ingestion of Dh-ATX is much more toxic than ingestion of ATX [44]. The toxicities of HATX and of ATX are similar [59]. In addition, ATX is known to bioaccumulate [60]. ATXs reached readings above the WHO recommended guideline value for bathing water of 60 µg/L in five samples of two different lakes which must be considered a human health threat. In one of the lakes (Lake Ostersee), the toxin consisted of the components ATX, Dh-ATX, and HATX. The samples from lake Reservoir Mandichosee did not contain HATX. The maximum ATXs content of almost 45,000 µg/L exceeded the WHO guideline value by 750 times and consisted of 99% Dh-ATX. Like for SXT, it is important to note that the WHO guideline values are only valid for open-water samples. For the benthic samples an uptake of such a high toxin dose would probably only be possible by direct ingestion of mat material, as the overlying water column was mostly clear of toxins.

For comparison, in mice, the 50% lethal dose (LD_50_) for oral intake of Dh-ATX is 8 mg/kg body weight [44]. Consequently, the high toxin concentration occurring at Reservoir Mandichosee due to benthic cyanobacteria, equivalent to almost 45 mg/L of pure Dh-ATX, can be classified as highly toxic. No cyanobacteria other than *Tychonema* were detected in this sample. It can therefore be assumed that the ATXs producer was definitely the genus *Tychonema*. This genus has already been studied in the past in connection with ATXs production in Reservoir Mandichosee and the River Lech [18,19]. In Lake Ostersee, the ATXs producers were likely *Kamptonema*, *Tychonema* and *Oscillatoria*.

In total, over 200 MCs variants are known, with MC-LR, MC-RR, MC-YR, and MC-LA being the most common [61]. The microcystin variant with the highest known toxicity is MC-LR, followed by MC-YR and MC-RR [62,63]. However, the toxicity of most microcystin variants (>180) is unknown.

The WHO guideline value for MCs in bathing waters of 24 µg/L was only exceeded in four open-water samples tested. In Reservoir Eixendorfersee, *Microcystis* sp., and in Aqua park Moosburg and Lake Pelhamersee, *Planktothrix rubescens* were clearly identified as MCs producers. In Lake Ebingersee, the MCs producer was probably also the genus *Microcystis*. This assumption is further supported by the microcystin variants present in the samples. Thus, in the Lake Eixendorfersee and Lake Ebingersee samples, the variants MC-RR, MC-YR and MC-LR produced by *Microcystis* dominated, whereas in the Aqua park Moosburg and Lake Pelhamersee, the variant [Asp^3^]-MC-RR produced by *Planktothrix* prevailed [64].

## 5. Conclusions

In addition to the previously known occurrences of planktic cyanobacteria, benthic cyanobacteria were also detected in many of the studied lakes. These could be documented especially in lakes with low nutrient levels. The benthic cyanobacteria were mainly filamentous forms of the orders Nostocales and Oscillatoriales. The representatives of the genera found in the benthic and open-water samples are theoretically capable of producing ATXs, CYNs, MCs and STX. The detected cyanotoxins were ATXs, MCs, and STX. ATXs were most frequently detected in the benthic samples and reached the highest concentrations, whereas MCs was the predominant cyanotoxin in the open-water samples.

Based on the results of the investigations, it was possible to identify lakes where a risk for toxic benthic or planktic cyanobacterial blooms already exists or will arise in the future.

In addition to MCs, the cyanotoxins ATXs and STX must also be expected in Bavarian lakes. Future studies should place a greater focus on benthic cyanobacterial occurrences, since the data are still very limited. Even though mass occurrences of toxin-producing benthic cyanobacteria have so far only been observed in one reservoir (Reservoir Mandichosee), their occurrence must also be expected in other waters. In this context, oligotrophic waters in particular should be studied in the future. Further investigations should particularly focus on the occurrence of the neurotoxin ATX and its variants.

## Figures and Tables

**Table 1:** Taxa composition of the benthic samples. The dominant taxon is marked with an asterisk, respectively. “Other” = taxa which accounted for less than 2% of the sequences were grouped together. A list of lake names and their abbreviations can be found in the Supplementary Materials (Table S1).

Taxon **	BHN	STM	PIL	SLI	DEC	DIT	GOS	GUG	IGL	KBR	KLO	MAN
*Anabaena*	-	-	-	-	-	+	-	-	-	-	-	-
*Aphanizomenon*	-	-	+	-	-	-	-	-	-	-	-	-
Caenarcaniphilales **	-	-	-	-	-	-	-	-	-	+	-	-
*Calothrix*	-	-	+	-	+	-	-	-	-	-	+	-
*Candidatus obscuribacter*	-	-	-	-	+	-	-	-	-	-	-	-
*Cyanobium*	+	-	+	+	+ *	+	+	+	+	+ *	+	+
*Cyanomargarita*	-	-	-	+ *	-	-	-	-	-	-	-	-
*Cyanothece*	-	-	-	-	-	-	-	-	-	+	-	-
*Dactylothamnos*	-	-	-	-	-	-	-	-	-	-	+	-
*Geitlerinema*	-	-	-	-	-	-	-	-	-	-	-	+
*Geminocystis*	-	-	-	-	-	-	-	-	-	+	-	-
*Gloeobacter*	-	-	+	+	-	-	-	-	-	-	-	-
*Kamptonema*	-	+ *	-	-	-	+	+	-	-	-	-	-
*Leptolyngbya*	-	-	-	-	+	-	-	-	+	-	+ *	-
Leptolyngbyaceae **	-	-	+	+	+	-	-	-	+ *	+	+	-
*Limnothrix*	-	-	-	-	+	-	-	-	-	-	-	-
*Microcoleus*	-	-	-	-	-	+	-	-	-	-	+	-
Microcystaceae **	-	-	-	-	-	-	-	-	-	+	-	-
*Microcystis*	-	-	+	-	-	-	-	-	-	-	-	-
*Neolyngbya*	-	-	-	-	-	-	+	+ *	-	-	-	-
*Nodosilinea*	-	-	-	-	+	-	-	-	-	+	+	-
*Oscillatoria*	+	-	-	-	-	+ *	+ *	-	-	-	-	-
Phormidiaceae **	-	-	-	-	-	-	-	+	-	-	-	-
*Phormidium*	+	-	-	-	-	-	-	-	-	-	-	-
*Planktothrix*	-	+	-	-	-	-	-	+	-	+	-	-
*Pleurocapsa*	-	-	+ *	-	-	-	-	-	-	-	-	-
*Prochlotothrix*	+	-	-	-	-	-	-	-	-	-	-	-
*Pseudanabaena*	+	-	-	-	+	-	-	-	-	+	-	+
Pseudanabaenaceae **	-	-	+	+	-	-	-	-	-	-	-	-
*Scytolyngbya*	-	-	-	-	-	-	-	-	-	-	+	-
*Scytonema*	-	-	-	-	-	-	-	-	-	-	+	-
*Sericytochromatia*	-	-	-	-	-	-	-	-	-	+	-	-
*Snowella*	-	-	-	-	-	+	-	-	-	-	-	-
*Synechococcus*	-	-	-	+	-	-	-	-	-	-	-	-
*Synechocystis*	-	-	+	-	-	-	-	-	-	-	-	-
*Tenebriella*	+	-	-	-	-	-	+	-	-	-	-	-
*Tychonema*	-	-	-	-	-	+	+	-	-	+	-	+ *
Other	+ *	+	+	+	+	+	+	+	+	+	+	+
Total number of genera	6	3	9	6	8	7	6	4	3	11	9	4

* dominant taxon; ** for individual taxa that could not be classified to genus level, the family or order is provided instead (represented by ‘+’).

## Data Availability

The data presented in this study are openly available in the Open Science Framework (OSF) at DOI 10.17605/OSF.IO/JC4F8, reference number [65].

## Supplementary Materials

The following supporting information can be downloaded at: https://www.mdpi.com/article/10.3390/toxics11080643/s1, Table S1: List of abbreviations of the lake names, Table S2: Genus composition in the open-water samples (in water bodies with benthic cyanobacteria findings). The dominant taxa are marked with an asterisk. The open-water samples of BHN, STM and GOS were not sequenced, Table S3: Results of the toxin measurements of all samples. MCs = sum of microcystin variants, ATXs = sum of anatoxin variants, SXT = saxitoxin, CYN = sum of cylindrospermopsin variants, n.d. = not detectable, “-” = not measured. MCs, ATXs and CYNs have been quantified with LC-MS/MS. SXT has been quantified using ELISA.
