# Supplementary material for: Occurrence, Distribution and Toxins of Benthic Cyanobacteria in German Lakes
Source: Toxics. 2023 Jul 25;11(8):643. doi: 10.3390/toxics11080643 (PMC10458227; doi:10.3390/toxics11080643)
Supplement: Supplementary file 1 [file toxics-11-00643-s001.zip › toxics-2494360-supplementary.pdf]

Supplementary Materials: Occurrence, Distribution and Toxins of Benthic Cyanobacteria in German Lakes

Franziska Bauer, Immanuel Wolfschlaeger, Juergen Geist, Jutta Fastner, Carina W. Schmalz and Uta Raeder

Table S1. List of abbreviations of the lake names.

ID	lake
**ALM**	Lake Altmuehlsee
**AQM**	Aqua Park Moosburg
**BHN**	Burgheim Natural Pond
**BIN**	Quarry Pond Ingolstadt
**BKW**	Lake Bergknappweiher
**DEC**	Lake Dechsendorferweiher
**DIT**	Lake Dietlhofersee
**EBS**	Lake Ebingersee
**EBW**	Lake Ebenhauserweiher
**EGG**	Lake Egglburgersee
**EHS**	Lake Ebertshausenersee
**EIX**	Reservoir Eixendorfersee
**FRI**	Lake Friedlsee
**FRS**	Lake Freudensee
**GOS**	Lake Ostersee
**GUG**	Lake Guggenbergersee
**IGL**	Lake Igelsbachsee
**KBR**	Lake Brombachsee
**KLO**	Lake Klostersee
**KRU**	Lake Russweiher
**LSP**	Reservoir Liebenstein
**MAE**	River Maedelelech
**MAN**	Reservoir Mandichosee
**OIM**	Reservoir Oberilzmuehle
**PEL**	Lake Pelhamersee
**PIL**	Lake Pilsensee
**ROD**	Lake Rothdach
**ROT**	Lake Rottachsee
**SIL**	Lake Silbersee
**SIM**	Lake Simssee
**SLI**	Lake Schliersee
**STM**	Lake Stoibermühle
**TAC**	Lake Tachingersee
**WAG**	Lake Wagingersee

Table S2. Genus composition in the open water samples (in water bodies with benthic cyanobacteria findings). The dominant taxa are marked with an asterisk. The open water samples of BHN, STM and GOS were not sequenced.

	BHN **	STM **	PIL	SLI	DEC	DIT	GOS **	GUG	IGL	KBR	KLO	MAN
Aliterella			-	-	-	-		-	-	-	-	+
Aphanizomenon			+	-	+	+*		-	-	+	+*	+
Calothrix			-	-	+	-		-	-	-	-	-
Cuspidothrix			-	-	+	-		-	-	-	-	-
Cyanobium			+*	+*	+	+		+*	+*	+	+	+
Dactylo-thamnos			-	-	-	-		-	-	-	-	+
Leptolyngbya			-	-	-	-		-	-	-	-	+
Limnoraphis			+	-	-	-		-	-	-	-	-
Microcystis			-	+	-	+		+	+	+*	+	-
Planktothrix			-	-	+*	-		-	-	-	-	-
Prochlorothrix			-	+	-	-		-	-	-	-	-
Pseudanabaena			-	+	-	-		-	-	-	-	+
Tychonema			-	-	-	-		-	-	-	-	+*
Woronichinia			-	-	-	-		-	-	+	-	-
Others			+	+	+	+		+	+	+	-	+
**Total number of genera**			**4**	**5**	**6**	**4**		**3**	**3**	**5**	**3**	**8**

*dominant taxon.
**the pelagic samples of BHN, STM and GOS were not sequenced.

Table S3. Results of the toxin measurements of all samples. MCs = sum of microcystin variants, ATXs = sum of anatoxin variants, SXT = saxitoxin, CYN = sum of cylindrospermopsin variants, n.d. = not detectable, "-" = not measured. MCs, ATXs and CYNs have been quantified with LC-MS/MS. SXT has been quantified using ELISA.

lake	sampling date	habitat	MCs [µg/L]	ATXs [µgL]	SXT [µg/L]	CYN [µg/L]
**ALM**	2021-07-21	planktic	n.d.	0.0	0.0	n.d.
**ALM**	2021-08-18	planktic	n.d.	0	-	n.d.
**ALM**	2021-09-22	planktic	n.d.	0	-	n.d.
**ALM**	2021-10-28	planktic	n.d.	0	-	n.d.
**ALM**	2021-12-08	planktic	n.d.	0	-	n.d.
**ALM**	2022-01-19	planktic	n.d.	0	-	n.d.
**ALM**	2022-03-23	planktic	n.d.	-	-	n.d.
**AQM**	2021-12-15	planktic	181	n.d.	0.0	n.d.
**AQM**	2022-01-17	planktic	4	n.d.	-	n.d.
**AQM**	2022-03-28	planktic	5	n.d.	n.d.	n.d.
**KBR**	2021-07-21	benthic	8	0	n.d.	n.d.
**KBR**	2021-07-21	planktic	1	0	n.d.	n.d.
**KBR**	2021-08-18	planktic	n.d.	0	-	n.d.
**KBR**	2021-09-22	planktic	0.0	0	-	n.d.
**KBR**	2021-10-28	planktic	0.0	0	-	n.d.
**KBR**	2021-12-08	planktic	n.d.	0	-	n.d.
**KBR**	2022-01-19	planktic	n.d.	0	-	n.d.
**KBR**	2022-03-23	benthic	n.d.	0.1	-	n.d.
**KBR**	2022-03-23	planktic	n.d.	0	-	n.d.
**BHN**	2021-09-14	benthic	2	2	-	n.d.
**BHN**	2021-09-14	planktic	0.3	0	n.d.	n.d.
**DEC**	2021-08-17	planktic	0.0	1	0.1	n.d.
**DEC**	2021-09-21	benthic	n.d.	0.3	0.6	n.d.
**DEC**	2021-09-21	planktic	n.d.	0.4	0.4	n.d.
**DEC**	2021-10-27	planktic	n.d.	0.2	0.1	n.d.
**DEC**	2021-12-07	planktic	n.d.	0	0.0	n.d.
**DEC**	2022-01-18	planktic	n.d.	0	0.0	n.d.
**DEC**	2022-03-22	benthic	0.4	0	0.0	n.d.

**DEC**	2022-03-22	planktic	n.d.	0	0.0	n.d.
**DEC**	2022-07-20	benthic	n.d.	0.2	0.1	n.d.
**DEC**	2022-07-20	planktic	n.d.	0.4	0.1	n.d.
**DIT**	2021-08-04	planktic	n.d.	0	n.d.	n.d.
**DIT**	2021-09-06	benthic	3	12	0.0	n.d.
**DIT**	2021-09-06	planktic	n.d.	0	n.d.	n.d.
**DIT**	2021-09-28	benthic	n.d.	0.5	n.d.	n.d.
**DIT**	2021-09-28	planktic	n.d.	0	0.03	n.d.
**DIT**	2021-11-24	benthic	n.d.	0.4	-	n.d.
**DIT**	2021-11-24	planktic	n.d.	0	-	n.d.
**DIT**	2021-12-15	benthic	n.d.	0.4	-	n.d.
**DIT**	2021-12-15	planktic	n.d.	0	-	n.d.
**DIT**	2022-01-12	benthic	n.d.	20	-	n.d.
**DIT**	2022-01-12	planktic	n.d.	0	-	n.d.
**DIT**	2022-02-08	benthic	n.d.	0.3	-	n.d.
**DIT**	2022-02-08	planktic	n.d.	0	-	n.d.
**DIT**	2022-03-24	planktic	n.d.	0	-	n.d.
**DIT**	2022-06-21	benthic	n.d.	0.1	n.d.	n.d.
**DIT**	2022-06-21	planktic	n.d.	0.0	0.0	n.d.
**EBW**	2021-06-29	planktic	n.d.	0	n.d.	n.d.
**EBW**	2021-08-26	planktic	n.d.	0	-	n.d.
**EBW**	2021-09-14	benthic	0.7	0.2	-	n.d.
**EBW**	2021-09-14	planktic	n.d.	0	-	n.d.
**EBW**	2021-11-23	planktic	n.d.	0	-	n.d.
**EBW**	2022-01-11	planktic	n.d.	0	-	n.d.
**EBW**	2022-02-08	planktic	n.d.	0	-	n.d.
**EBW**	2022-03-21	planktic	n.d.	0	-	n.d.
**EBS**	2021-07-20	planktic	45	0	n.d.	n.d.
**EBS**	2021-08-17	benthic	0.4	0	n.d.	n.d.
**EBS**	2021-08-17	planktic	0.2	0	n.d.	n.d.
**EBS**	2021-09-21	benthic	8	0	n.d.	n.d.
**EBS**	2021-09-21	planktic	0.4	0	0.1	n.d.
**EBS**	2021-10-27	planktic	0.0	0	n.d.	n.d.
**EBS**	2021-12-07	planktic	1.5	0	-	n.d.
**EBS**	2022-01-18	planktic	n.d.	0	n.d.	n.d.
**EBS**	2022-03-22	planktic	n.d.	0	0.0	n.d.
**EGG**	2021-06-22	benthic	n.d.	0	0.03	n.d.
**EGG**	2021-06-22	planktic	n.d.	0	0.03	n.d.
**EGG**	2021-08-04	planktic	6.1	0	n.d.	n.d.
**EGG**	2021-09-06	planktic	1.4	0	n.d.	n.d.
**EGG**	2021-09-29	planktic	16.7	0	n.d.	n.d.
**EGG**	2021-11-24	planktic	0.0	0	n.d.	n.d.
**EGG**	2022-01-12	planktic	n.d.	0	n.d.	n.d.
**EGG**	2022-02-22	planktic	n.d.	0	-	n.d.
**EGG**	2022-03-24	planktic	n.d.	0	0.0	n.d.
**EIX**	2021-09-17	planktic	17780	0	-	n.d.
**GOS**	2021-11-03	benthic	0.4	81	0	n.d.
**GOS**	2021-11-03	benthic	0.3	9	0.0	n.d.
**GOS**	2021-11-03	planktic	n.d.	n.d.	n.d.	n.d.
**GUG**	2021-07-13	benthic	n.d.	0	0.0	n.d.
**GUG**	2021-07-13	planktic	n.d.	0.1	0.0	n.d.
**GUG**	2021-08-25	benthic	1.3	0	-	n.d.
**GUG**	2021-08-25	planktic	0.6	0	-	n.d.
**GUG**	2021-09-14	planktic	0.3	0	-	n.d.
**GUG**	2021-10-13	planktic	0.8	0	-	n.d.
**GUG**	2021-11-23	planktic	1.5	0	-	n.d.

**GUG**	2022-01-11	planktic	n.d.	0	-	n.d.
**GUG**	2022-03-21	benthic	n.d.	0	-	n.d.
**GUG**	2022-03-21	planktic	n.d.	0	-	n.d.
**IGL**	2021-08-18	planktic	n.d.	0	-	n.d.
**IGL**	2021-09-22	benthic	0.0	0	-	n.d.
**IGL**	2021-09-22	planktic	n.d.	0	-	n.d.
**IGL**	2021-10-28	planktic	n.d.	0	-	n.d.
**IGL**	2021-12-08	benthic	6.6	4	-	n.d.
**IGL**	2021-12-08	planktic	n.d.	0	-	n.d.
**IGL**	2022-01-19	planktic	n.d.	0	-	n.d.
**IGL**	2022-03-23	planktic	n.d.	0	-	n.d.
**IGL**	2022-07-21	planktic	n.d.	0.1	0.0	n.d.
**KLO**	2021-06-22	benthic	n.d.	0	0.0	n.d.
**KLO**	2021-06-22	planktic	n.d.	0	0.0	n.d.
**KLO**	2021-08-04	planktic	n.d.	0	n.d.	n.d.
**KLO**	2021-09-06	planktic	n.d.	0	0.0	n.d.
**KLO**	2021-09-29	benthic	0.5	0	0.0	n.d.
**KLO**	2021-09-29	planktic	n.d.	0	n.d.	n.d.
**KLO**	2021-11-24	planktic	n.d.	0	n.d.	n.d.
**KLO**	2021-12-15	planktic	n.d.	0	-	n.d.
**KLO**	2022-01-12	planktic	n.d.	0	-	n.d.
**KLO**	2022-02-22	benthic	0.2	0	n.d.	n.d.
**KLO**	2022-02-22	planktic	n.d.	0	n.d.	n.d.
**KLO**	2022-03-24	planktic	n.d.	0	0.0	n.d.
**MAN**	2021-06-30	benthic	n.d.	0.1	n.d.	n.d.
**MAN**	2021-06-30	planktic	n.d.	0.0	n.d.	n.d.
**MAN**	2021-07-27	planktic	n.d.	2	n.d.	n.d.
**MAN**	2021-09-07	benthic	n.d.	407	-	n.d.
**MAN**	2021-09-07	planktic	n.d.	4	-	n.d.
**MAN**	2021-09-28	benthic	n.d.	119	-	n.d.
**MAN**	2021-09-28	planktic	n.d.	0	-	n.d.
**MAN**	2021-11-23	benthic	n.d.	2	-	n.d.
**MAN**	2021-11-23	planktic	n.d.	0	-	n.d.
**MAN**	2021-12-15	benthic	n.d.	0.1	-	n.d.
**MAN**	2021-12-15	planktic	n.d.	0	-	n.d.
**MAN**	2022-01-11	benthic	n.d.	0	-	n.d.
**MAN**	2022-01-11	planktic	n.d.	0	-	n.d.
**MAN**	2022-02-08	benthic	n.d.	0	-	n.d.
**MAN**	2022-02-08	planktic	n.d.	0	-	n.d.
**MAN**	2022-03-21	benthic	n.d.	0	-	n.d.
**MAN**	2022-03-21	planktic	n.d.	0	-	n.d.
**MAN**	2021-07-27	benthic (a)	2	44971	n.d.	n.d.
**MAN**	2021-07-27	benthic (b)	n.d.	469	-	n.d.
**PEL**	2021-08-12	planktic	101	n.d.	n.d.	n.d.
**STM**	2021-08-26	benthic	n.d.	2	0.0	n.d.
**STM**	2021-08-26	planktic	n.d.	0	n.d.	n.d.
